# Mechanisms and Factors that Influence High Frequency Retroviral Recombination

**DOI:** 10.3390/v3091650

**Published:** 2011-09-09

**Authors:** Krista Delviks-Frankenberry, Andrea Galli, Olga Nikolaitchik, Helene Mens, Vinay K. Pathak, Wei-Shau Hu

**Affiliations:** 1 Viral Mutation Section, HIV Drug Resistance Program, National Cancer Institute at Frederick, Frederick, MD 21702, USA; E-Mails: frankenk@mail.nih.gov (K.D.-F.); vinay.pathak@nih.gov (V.K.P.); 2 Viral Recombination Section, HIV Drug Resistance Program, National Cancer Institute at Frederick, Frederick, MD 21702, USA; E-Mails: andrea.galli@hvh.regionh.dk (A.G.); nikolaio@mail.nih.gov (O.N.); 3 Copenhagen Hepatitis C Program, Department of Infectious Diseases, Copenhagen University Hospital, Hvidovre 2650, Denmark; 4 Department of Epidemic Diseases, Rigshospitalet, København 2100, Denmark; E-Mail: helene.mens@gmail.com

**Keywords:** recombination, retrovirus, HIV-1

## Abstract

With constantly changing environmental selection pressures, retroviruses rely upon recombination to reassort polymorphisms in their genomes and increase genetic diversity, which improves the chances for the survival of their population. Recombination occurs during DNA synthesis, whereby reverse transcriptase undergoes template switching events between the two copackaged RNAs, resulting in a viral recombinant with portions of the genetic information from each parental RNA. This review summarizes our current understanding of the factors and mechanisms influencing retroviral recombination, fidelity of the recombination process, and evaluates the subsequent viral diversity and fitness of the progeny recombinant. Specifically, the high mutation rates and high recombination frequencies of HIV-1 will be analyzed for their roles in influencing HIV-1 global diversity, as well as HIV-1 diagnosis, drug treatment, and vaccine development.

## 1. Introduction

Retroviridae is a family of RNA viruses that include an integrated DNA phase in their replication cycle. Several members of this family are viral human pathogens such as human immunodeficiency virus type 1 and type 2 (HIV-1 and HIV-2). One of the hallmarks of retroviral replication is the high frequency of recombination that can assort mutations in the viral genome to increase viral population diversity. High variation increases the probability of the population to survive, as it is likely that some proportion of the viral population will be able to escape the changing environmental selection pressures.

Most retroviruses exhibit very high population diversity; one of the best examples is the variation observed with HIV-1. HIV-1 can be divided into four groups, M, O, N, and P; however, 95% of the HIV-1 infections worldwide are caused by group M. Although group M virus was first transmitted into the human population by a single zoonotic event from SIVcpz, a simian immunodeficiency virus that infects chimpanzees [1], group M has diverged tremendously in the human population and therefore can be further divided based on sequence identity into several subtypes (A, B, C, D, F, G, H, J, and K) [2] and intersubtype recombinants. Different group M subtypes vary in their prevalence and distribution; for example, subtype C variants are the most widespread, accounting for 48% of all HIV-1 infections globally, whereas most HIV-1 infections in the Americas and Western Europe are caused by subtype B variants [3]. Generally, variants within the same subtype have more than 90% sequence identity whereas variants from different subtypes have between 80–90% sequence identity in their genomes. One of the major mechanisms generating this high diversity is frequent recombination, which can quickly distribute existing sequences and generate variants with different assortments of polymorphisms in the viral population. Recombination can occur within the same subtype, known as intrasubtype recombination, or between different subtypes, known as intersubtype recombination. Recombination can also occur between different HIV groups, albeit rarely. This review focuses on the mechanisms and factors that affect retroviral recombination, and how recombination influences HIV-1 diagnosis, treatment, and vaccine development.

### 1.1. Retroviral Replication Cycle

The detailed mechanisms of retrovirus replication are complex and involve intricate interactions with the host cell machinery at multiple steps [4]. Retroviruses package two copies of full-length viral RNAs into each particle, and each mature particle contains three virally encoded enzymes: protease, reverse transcriptase (RT) and integrase. RT uses the viral RNA as a template to synthesize a double-stranded DNA copy of the viral genome. This process, known as reverse transcription, occurs in the target cells for all retroviruses except spumaviruses [5]. The newly synthesized viral DNA integrates into the host cell genome, a step that is mediated by the viral enzyme integrase, to form a provirus. Host cell machinery transcribes the provirus to generate viral RNAs; some of the viral RNAs are spliced whereas others remain full-length. Viral RNAs are then exported out of the nucleus and used as templates for the translation of viral proteins. Newly synthesized viral proteins, together with two copies of the full-length viral RNA, assemble into viral particles to be released from the host cell.

### 1.2. Reverse Transcription of the Viral RNA into DNA

**Figure 1.**
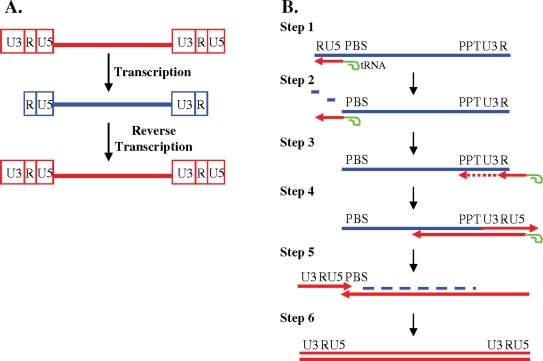
Reverse Transcription. (**A**) General structures of retroviral DNA and RNA genomes; (**B**) Processes required to complete the synthesis of viral DNA from the viral RNA genome. Red, DNA; blue, RNA; green, tRNA primer; arrow, direction of DNA synthesis. RNase H degradation, blue dashes.

Although RT is the only enzyme required for synthesizing DNA from an RNA template *in vitro*, reverse transcription of the viral genome *in vivo* is connected to many other steps in early viral infection such as uncoating [6]. Figure 1 shows the complicated, but elegant process of reverse transcription [4]. A provirus generally has two identical blocks of sequences at each end known as long terminal repeats (LTRs) (Figure 1A). LTRs can be further divided into three regions, unique 3′ region (U3), repeat (R), and unique 5′ region (U5). As shown in Figure 1A, the sequence of the viral RNA is shorter than the proviral DNA, lacking both the 5′ U3 region and the 3′ U5 region. Since the viral promoter is located in the 5′ U3 region, the expression of the viral DNA is dependent upon the process of reverse transcription to reconstitute full-length LTRs.

Reverse transcription involves at least two obligatory template switching events, namely, minus-strand and plus-strand DNA transfer (Figure 1B). Reverse transcription initiates near the 5′ end of the viral RNA using a host cell tRNA primer, which is partially annealed to a region of viral RNA based on complementarity, termed the primer binding site (PBS) (Step 1). Since the viral RNA packaged into the virion is plus sense, the first strand of DNA synthesized is referred to as the minus strand. RT, which is also a DNA polymerase, copies a short stretch of the viral RNA including the 5′ U5 and 5′ R region. In addition to the DNA polymerase activity, RT also has RNase H activity, which specifically degrades RNA in an RNA:DNA hybrid. Degradation of the RNA that was used as template to synthesize the minus-strand DNA, exposes the newly synthesized 5′ R region (Step 2), which can form base-pairing with the R region at the 3′ end of the viral genome, completing the first template-switching event known as minus-strand DNA transfer. It has been shown that during this step, the minus-strand DNA can transfer either to the same RNA molecule (intramolecular transfer) or to the copackaged RNA (intermolecular transfer) [7–9]. RT then continues copying the U3 located at the 3′ end of the viral RNA (Step 3). Once minus-strand DNA synthesis continues past a region called the polypurine tract (PPT), which is resistant to RNase H cleavage, RT makes a specific cleavage between the PPT and 3′ U3. RT uses the PPT as a primer to initiate plus-strand DNA synthesis and copies the 3′ LTR (U3, R and U5) along with a portion of the tRNA primer (Step 4). The plus-strand DNA synthesis in many retroviruses is discontinuous and can initiate from more than one position; some viruses, such as HIV-1, contains a second PPT located in the middle of the genome termed central PPT. Using the short complementarity between the plus-strand DNA and the PBS region on the minus-strand DNA, RT makes a second obligatory switch, termed plus-strand DNA transfer (Step 5); although shown as a linear model, it is likely that the two ends of the nucleic acid complex are in close proximity during plus-strand DNA transfer. Both plus-strand and minus-strand DNA synthesis then continue to complete reverse transcription (Step 6).

### 1.3. Retroviral Recombination Models

In order to complete reverse transcription, RT must undergo two obligatory template switches. Based on the natural ability of RT to switch templates, a model for retroviral recombination was proposed known as the forced copy choice model [10]. This model proposes that due to the presence of breaks found in the packaged viral RNA, RT will switch to the copackaged RNA when it encounters a break in the RNA, and use it as a template for DNA synthesis. As a result, a recombinant DNA molecule will be generated that contains portions of the genetic information from each copackaged RNA. This model was later modified to be known as the dynamic copy choice model [11], which proposes that template switching is influenced by a balance between the polymerase and RNase H activity of RT; during minus-strand DNA synthesis, polymerase activity synthesizes the DNA whereas RNase H activity degrades the RNA in the RNA:DNA hybrid. This process is dynamic, and when the newly synthesized DNA in the RNA:DNA hybrid becomes exposed, it is more likely to switch to the other copackaged RNA and use it as a template for DNA synthesis. This model is described in more detail in Section 2.

The forced copy choice and dynamic copy choice models describe recombination that occurs during minus-strand DNA synthesis; the strand displacement assimilation model has also been proposed to depict recombination during plus-strand DNA synthesis [12]. This model was based upon the observation that in avian retroviruses, such as avian sarcoma leukosis virus, plus-strand DNA synthesis is discontinuous and synthesized in many pieces. This model posits that some of the short pieces of plus-strand DNA can dissociate and anneal to the minus-strand DNA synthesized using the copackaged RNA as a template. After DNA repair that preserves the genetic information on the plus-strand DNA, a recombinant can be generated.

### 1.4. Requirements for the Generation of Observable Recombination Events

The high frequency of retroviral recombination occurs during reverse transcription of the copackaged viral RNA, and not during coinfection between two different viruses [13]. A dually-infected cell that contains two proviruses can generate viral particles that contain two copies of RNA from parent 1 or two copies of RNAs from parent 2 (homozygous particles), or one copy of RNA from parent 1 and one copy of RNA from parent 2 (heterozygous particles). Although recombination can occur in both homozygous and heterozygous particles, only the progeny of heterozygous particles can generate a recombinant that is genotypically different than the two parents [13]. Therefore, multiple requirements must be met to generate a recombination event that can be identified: (1) a cell must be infected by more than one virus; (2) both proviruses must express their genomes; (3) RNAs from the coinfected proviruses must be copackaged; (4) RT must switch templates between the copackaged RNAs to generate a recombinant DNA copy; and (5) the recombinant DNA integrates and expresses its genome. Although multiple steps are required to generate a recombinant, retroviruses still have one of the highest recombination rates. The impact of this high recombination potential can be demonstrated in the current HIV-1 epidemic as a large proportion of the currently circulating strains of HIV-1 are known recombinants.

## 2. Template Switching During Reverse Transcription

Reverse transcription of the retroviral genome requires RT to undergo two obligatory template switching (strand transfer) events to complete viral DNA synthesis. This requirement leads to the proposal that RT evolved to select for low template affinity and low processivity in order to complete reverse transcription. RT is estimated to dissociate from the template during reverse transcription at least 8 times for a 10-kb viral genome [14]. Specifically for HIV-1, it is estimated that 3–12 template switches occur per genome per replication cycle [15–17]. Since retroviruses package two copies of their genome into the viral particle, template switches that occur can either be intermolecular, resulting in homologous or nonhomologous recombination [18–20], or intramolecular, resulting in deletions, deletions with insertions, and insertions and duplications [14,21,22]. However, if RT reassociates back onto the same complex at the same position, then no recombination occurs. In this section, we will discuss factors that influence template switching, as measured through *in vivo* direct repeat deletions and *in vitro* biochemical studies, in simple retroviruses such as spleen necrosis virus (SNV) and murine leukemia virus (MLV) as well as complex retroviruses such as HIV-1.

### 2.1. In Vivo Direct Repeat Deletion Reveals that Template Switching Occurs Frequently During Reverse Transcription and is Dependent Upon the Size and Distance Between the Direct Repeats

Since the early 1980s it has been reported that directly repeated sequences in both viral genomes and viral vectors are unstable [23–26]. Specifically, direct repeats in the enhancer sequences of the viral LTR (50–75 bp in length) have been shown to vary in copy number and subsequently alter viral expression [27,28]. An SNV retroviral vector containing a 110-bp direct repeat was found to delete at a frequency of 41% in a single cycle assay [29], and it was hypothesized that the RT and the nascent strand dissociate from the template during reverse transcription, reassociating with a homologous section of template, resulting in deletion of the directly repeated sequence and any intervening sequences [21].

**Figure 2.**
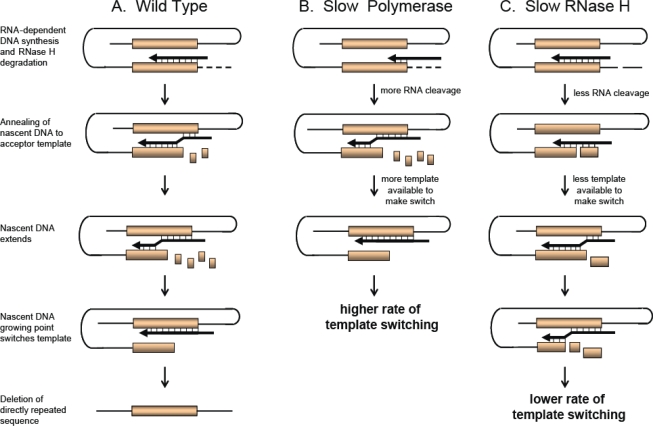
Dynamic Copy Choice Model. Shown are two direct repeats (orange box) undergoing an intramolecular template switch during minus-strand DNA synthesis (thick black arrow and line) resulting in deletion of the directly repeated sequence. RNA strand (thin black line) is depicted as “looped”, aligning the homologous directly repeated sequences during reverse transcription. (**A**) Direct repeat deletion by wild type reverse transcriptase (RT). During reverse transcription, degradation of the RNA template allows the newly synthesized DNA strand to anneal to the acceptor template (upstream direct repeat). The nascent DNA growing point switches templates, reverse transcription continues and one of the direct repeats is deleted; (**B**) RTs with a slow polymerase will increase template switching; (**C**) RTs with a slow RNase H will decrease template switching.

A model for direct repeat deletion and recombination [21,30,31] is shown in Figure 2A. The model shows that during RNA-dependent DNA synthesis, dissociation of RT and the growing nascent strand occurs due to RTs inherent low affinity to the template and the resulting minimal base pairing of the growing strand with the RNA template from RNase H degradation. RT and the growing strand can either reassociate with the upstream directly repeated sequence on the same RNA strand (intramolecular; shown in Figure 2) or the copackaged RNA strand (intermolecular), resulting in deletion of one of the direct repeats. Although shown with direct repeats, this model also describes recombination during DNA synthesis when the two homology blocks shown in Figure 2 are from two different RNAs.

**Figure 3.**
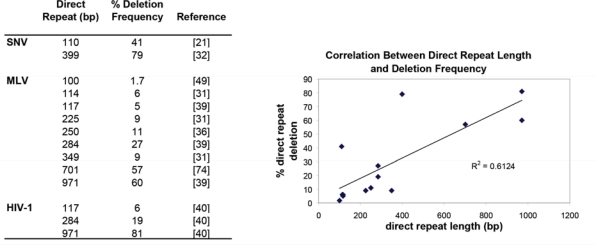
Relationship Between Direct Repeat Size and Deletion Frequency. Left: Summary table showing the reported deletion frequencies of direct repeats from MLV, SNV and HIV-1; right: correlation between the size of the direct repeat and the frequency of deletion.

Direct repeat deletions occur mostly through intramolecular template switches [18] and the rates of template switching are similar during RNA- and DNA-dependent DNA synthesis as measured by direct repeat deletion of a 400-bp direct repeat in an SNV viral vector system [32]. The frequency of direct repeat deletions has been shown to be dependent upon the size of the direct repeats for both simple and complex retroviruses. Figure 3 summarizes the reported direct repeat deletion frequencies and shows in general that the larger the direct repeat, the higher the frequency of direct repeat deletion. Furthermore, the deletion frequencies are similar amongst the tested retroviruses suggesting that the MLV, SNV and HIV-1 RTs share some properties, such as template affinity and processivity, which determine the frequencies of RT template switching.

Overall, these results suggest that longer lengths of homology are one of the driving forces that influence a successful template switch. This was first suggested by Zhang and Temin that the efficiency of recombination is dependent upon the length of template identity [33]. Confirmation of this idea through an experiment using direct repeats was shown by keeping the direct repeat length constant, but varying the percent sequence similarity between the two direct repeats. Decreasing the percent sequence similarity to 95% dropped the deletion frequency ∼2-fold, to 90% dropped the deletion frequency 4-fold, and anything lower than 82% homology dropped the deletion frequency to less than 1% [34]. Essentially, the experiment was also measuring non-homologous recombination and confirmed that nonhomologous recombination is inefficient, which was previously measured to be at least 1000-fold lower than the rate of homologous recombination [13,35].

Interestingly, increasing the distance between two direct repeats actually increases the frequency of deletion. Direct repeat deletion in an MLV vector in which a 701-bp direct repeat was separated by distances from 100 bp to 3.5 kb, resulted in high deletion frequencies (>90%) for distances >1500 bp [31]. This result provides insight into the organization of the RNA during reverse transcription indicating that large stretches of template are available for template switching upstream of the growing reverse transcription complex.

### 2.2. Frequency of Direct Repeat Deletion in Vivo Is Influenced by Nucleotide Pools and Mutations in RT

The frequency of direct repeat deletion is not only dependent upon the template itself, but can also be influenced by factors that affect RT activity. Drugs or mutations that affect the balance between polymerization and/or RNase H activity of RT can influence template switching. The dynamic copy choice model shown in Figure 2 demonstrates that RTs with a slow polymerase will have a higher rate of template switching, while RTs with lower RNase H activity will have lower rate of template switching [11,36,37]. By slowing down polymerization, more efficient degradation of the template strand occurs behind the growing nascent DNA strand providing more time for hydrogen bonding to occur between the acceptor template and the nascent DNA. This results in an increase in template switching (Figure 2B). In the case where RNase H activity is reduced, less nascent DNA is available to hydrogen bond with the template strand to facilitate a template switch, resulting in a reduced frequency of template switching (Figure 2C).

Support for this model has been validated by a number of studies. To induce RT to slow down polymerization, cells can either be treated with hyrdoxyurea (HU) to upset cellular nucleotide pools or treated with the nucleoside analog AZT to severely slow down polymerization. As expected, HU treatment in general showed a 2-fold increase in direct repeat deletion for both simple and complex retroviruses: SNV, 2.1-fold [38,39], MLV, 1.7–2.7-fold [11,36,39], and HIV-1, 1.8- 2.3-fold [37,40]. AZT treatment was also shown to increase direct repeat deletion in a dose dependent manner. The deletion frequency of a 250-bp direct repeat was shown to increase from 16% to 40% with increasing concentrations of AZT [41]. It was hypothesized that incorporation and excision of AZT-monophosphate slows down DNA synthesis, and this in turn increases template switching. This was also confirmed by analysis of a 117-bp direct repeat from an HIV-1 vector in which the presence of AZT caused a 1.6-fold increase in the deletion frequency [40]. RNA structure can also create pause sites and slow down polymerization resulting in an increase in template switching [42–48]. In MLV, a stem-loop structure placed between a 110-bp direct repeat increased deletion frequencies up to 5-fold [49]. Furthermore, studies using a 61-bp direct repeat separated by stem loops, showed deletion frequencies increased from 5% up to 47%, as the stability of the stem loop increased [50]. Lastly, even nucleotide sequence, for example, homopolymeric runs, can increase template switching [21,29,51,52].

Mutations in RT that also directly affect the polymerase activity and slow down polymerization, as expected, increase template switching. Mutations in the MLV RT dNTP binding site, polymerase active site, and thumb domain, which affect RT processivity, were also shown to increase direct repeat deletion *in vivo* [36]. This was also proven to be true for HIV-1, as mutations near the polymerase active site or dNTP binding site, such as K65R, Q151N, and E89G, increased direct repeat deletion 3-, 6- and 3-fold, respectively [37]. Interestingly, these mutations are selected in response to drug therapy in patients, and these results show that mutations conferring drug resistance can lead to increases in template switching, thus influencing not only recombination, but also viral evolution. Lastly, viral proteins, such as nucleocapsid (NC), can also influence template switching. In MLV, NC mutants which decreased the overall RT processivity (*i.e.*, slowed down polymerization), also increased direct repeat deletion. Whereas, NC mutants which caused a decrease in template annealing, decreased direct repeat deletion, as expected [49].

The dynamic copy choice model also predicts that decreasing RNase H activity will lead to a decrease in template switching. Mutations in the MLV RNase H (S526A, Y598V, R657S) and HIV RNase H (H539N, D549N) known to affect RNase H activity were shown to decrease template switching, resulting in a 2-fold decrease in direct repeat deletion [36,37]. Experiments in which MLV virions were phenotypically mixed to have lower levels of RNase H, demonstrated lower levels of direct repeat deletion [53]. For HIV, mutational analysis showed that mutations in the RNase H primer grip that affected template binding, and subsequently decreased RNase H activity, also decreased direct repeat deletion [54]. Interestingly, RT mutations in the connection and RNase H domain acquired by HIV-1 treatment-experienced patients, which reduce RNase H cleavage, also show a decrease in direct repeat deletion [55,56]. Eight novel mutations in HIV-1, E312Q, G335C/D, N348I, A360I/V, V365I, and A376S were identified that contribute to AZT resistance and also decrease template switching [55,56].

### 2.3. In Vitro Studies Dissecting the Biochemical Nature of Template Switching

Template switching events have also been extensively studied *in vitro* using donor and acceptor oligonucleotide templates, and have mostly modeled recombination events occurring during minus-strand synthesis. The “Dock and Lock” *in vitro* model [57–59] for strand transfer complements the *in vivo* dynamic copy choice model. During RNA-dependent DNA synthesis, RNase H cleavage occurs behind the polymerizing RT exposing regions of single-stranded cDNA that are accessible to anneal (“dock”) to an acceptor RNA template. RT and the nascent cDNA can then switch templates (“lock”) and continue synthesizing on the acceptor RNA template. This model for invasion-driven transfer as expected is influenced by RT polymerization activity, RNase H activity, and RNA structure.

Detailed studies on the sites of strand invasion, show that the invasion sites onto the acceptor template normally occur at the base of a strong hairpin structure or a site where RT pausing was significant [42,58–61]. Pausing slows down polymerization and allows more RNase H cleavages to occur, enhancing the probability for the acceptor RNA and nascent DNA to interact and undergo a template switch [48,61–63]. Destefano *et al*. showed that strand transfer was 25% lower in the absence of a pause site, and decreasing dNTP concentrations in the reactions also lead to an increase in strand transfer [62]. Mutations in RT polymerase that affect dNTP binding, such as Q151N and V148I in HIV-1, also show enhanced strand transfer that is attributed to stalled DNA synthesis and thus slower polymerization [64]. RNA hairpin structures such as the dimerization initiation signal (DIS) have also been shown to promote strand transfer *in vitro* [65,66] and will be discussed in further detail in subsequent sections. Studies on DIS suggest that dimerization increases the local concentration of the nascent DNA and acceptor RNA template increasing the rate of strand transfer.

The “Dock and Lock” model is dependent upon RNase H cleavage and the removal of the short RNA oligos to provide access for strand invasion. *In vitro* assays have shown that MLV or HIV-1 RTs that are deficient in RNase H activity cannot undergo template switching [62,63] and fail to undergo minus strand transfer [67,68]. Strand invasion and subsequent strand transfer is thus dependent upon both polymerase-dependent and polymerase-independent RNase H cleavage. Pausing increases polymerase-dependent primary and secondary RNase H cleavages [69] helping to create an invasion site, while the excess of polymerase-independent RTs packaged per virion (50–100) degrade the remaining hybridized RNA fragments, which also helps to contribute to strand transfer [70–73]. Overall, the *in vitro* and *in vivo* models for strand transfer are consistent in showing that the two activities of RT, polymerase and RNase H, play a major role in influencing recombination events.

### 2.4. The Use of Directly Repeated Sequences for the Development of Viral Vectors for Gene Therapy and for Probing In Vivo Reverse Transcription

**Figure 4.**
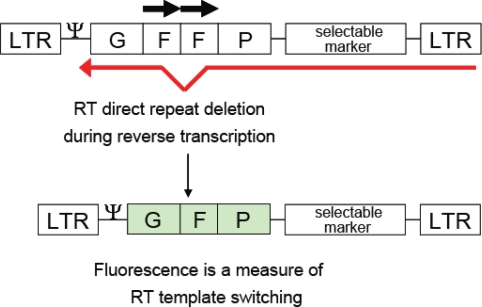
Single-cycle Vector to Monitor Viral Infection and the *In Vivo* Intramolecular Template Switching Rate. An HIV vector containing direct repeats (horizontal arrows) of the middle portion of the *gfp* gene (the “F” portion) is mobilized by co-transfection with vectors containing gag-pol and envelope. The frequency of a homologous template switch during reverse transcription is measured by reconstitution of the *gfp* gene. LTR, long terminal repeats.

Direct repeat deletion is highly precise and accurately deletes repetitive sequences. Because of this phenomenon, direct repeats can be used to make informative vectors for laboratory use to study recombination by analyzing intra- and intermolecular template switching. Gene sequences from selectable drug markers such as neomycin, herpes simplex thymidine kinase gene, and *lacZ* can be duplicated to create direct repeats, and used to determine if viral infection has taken place. Even more informative is the use of fluorescent gene sequences, such as the green fluorescent protein (GFP), to detect infection of MLV [36] or HIV-1 [37] in cell lines (Figure 4).

The power of these vectors can be extended further to also delete unwanted sequences after infection. For example, placing a selectable marker between the direct repeats will result in a virus lacking the selectable marker after infection [74,75]. Likewise, placing the packaging signal between the direct repeats can result in >99% efficient deletion [30,74,75], which would be useful for gene therapy and delivery of genes to target cells without further mobilization of the viral vector.

Overall, template switching is a dynamic process. A recent paper demonstrates that RT is able to slide, flip orientations, and dissociate from the template during reverse transcription [76]. These properties allow RT to complete the necessary “jumps” required during minus-strand strong-stop and plus-strand strong-stop transfer to complete reverse transcription, and undergo strand transfer events to reassort mutations and generate recombinants to ensure the survival of progeny virus.

## 3. Recombination: Rates, RNA Copackaging, and Fidelity

### 3.1. Recombination Rates in a Single Replication Cycle

**Figure 5.**
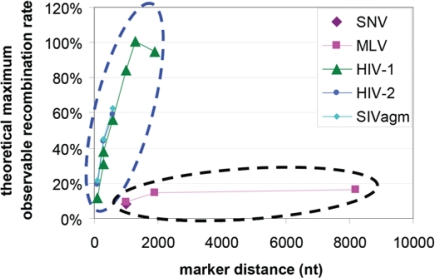
Retroviral Recombination Rates. X-axis, nucleotide distances between genetic markers; Y-axis, percent of theoretical maximum observable recombination rate [79]. Measured recombination rates from lentiviruses (dotted oval in blue) and gammretroviruses (dotted oval in black).

Retrovirus recombination rates were first measured in SNV and it was found that genetic markers separated by 1 kb recombined at 4% per replication cycle [13]. Similar measurements were made with MLV and it was found that markers separated by 1 kb, 1.9 kb, and 7.1 kb recombined at 4.7%, 7.4%, and 8.2%, respectively, per replication cycle [77]. Recombination rates were also measured in HIV-1 using the same template sequences as the MLV study and it was found that the recombination rates per replication for markers separated by 1 kb, 1.3 kb, and 1.9 kb were 42.4%, 50.4%, and 47.4%, respectively [78]. Using a different set of genetic markers, it was concluded that SIVagm and HIV-2 also recombine at high rates similar to HIV-1 [79,80]. Since the various assays used above generate rates that may not be directly comparable, we have converted the measured rates into percent of theoretical maximum measurable rates [79] as shown in Figure 5. These results point to the conclusions that lentiviruses (HIV-1, HIV-2, and SIVagm) appear to recombine more frequently than gammaretroviruses (SNV and MLV).

The cause of these differences in recombination frequencies is puzzling because RTs from SNV, MLV, and HIV-1 have similar template switching rates as mentioned above. This is further supported by the observation that multiple crossovers are often observed in SNV and MLV recombination, even though such events should be exceedingly rare based on their recombination rates [7,81]. Similarly, when examining MLV proviruses that are known to contain sequences from two different parents, frequent recombination can be detected elsewhere in the genomes [82]. These observations led to the conclusion that in SNV and MLV, recombination only occurs in a distinct retroviral population [18]; however, the nature of this population remains to be fully elucidated. Recent studies suggest that the efficiency of the RNA derived from two separate proviruses to copackage into the same particle has a significant impact on the observed recombination rates (discussed in detail below), providing insight into a possible mechanism that could contribute to the observed different recombination rates.

### 3.2. RNA Copackaging and Its Impact on Recombination Rates

The recombination rates discussed in the previous section (Figure 5) were measured using viruses derived from the same strain or molecular clones. However, recombination rates can be significantly lower between HIV-1 variants from different subtypes [83]. Subsequent analyses revealed that a 6-nt palindromic sequence in the 5′ untranslated region termed dimerization initiation signal (DIS) [84–86] plays a major role in determining the recombination potential of two HIV-1 variants [83,87,88]. Two variants with different DIS palindromes recombine less frequently than two variants with the same DIS palindrome. HIV-1 from various subtypes can have different DIS sequences; for example, most subtype B HIV-1 variants have GCGCGC in their DIS, whereas most subtype C variants have GTGCAC in their DIS [89].

Viral RNA dimerizes, or selects its copackaged partner, prior to encapsidating into particles [88,90]. Experimental evidence suggests that the two viral RNAs initiate their interactions by forming base-pairing in the palindromic DIS sequences [91,92]. By labeling the RNA in the virion at the single RNA level, it was shown that RNAs derived from two separate HIV-1 genomes can dimerize very efficiently when they contain the same DIS palindrome [93]. Assuming equal expression and random assortment, the theoretical distribution of particles containing two RNAs from parent 1, one RNA from each parent virus, and two RNAs from parent 2 is 25%:50%:25% (Hardy-Weinberg Equilibrium). It was shown that when the two parent RNAs were expressed at similar levels and contained the same GCGCGC palindromes in their DIS, heterozygous particles were generated ∼45% of the time, demonstrating that RNA from different viruses are packaged close to random distribution [93]. However, when the two parent viruses contained different DIS palindromes, for example, one had GCGCGC and the other GTGCAC, these RNAs were copackaged together far less frequently (∼20%) [93]. Furthermore, recombination rates were significantly lower with HIV-1 containing different DIS palindromes [83]. In another experiment in which two HIV-1 RNAs were modified to contain nonpalindromic DIS sequences that still could interact with each other, for example, one virus has CCCCCC and the other GGGGGG, copackaging of the two RNAs increased beyond that predicted by random assortment (from ∼45% to ∼70%), and the recombination rate also increased beyond that of two wild-type viruses containing the same palindromes [88]. Together, these experimental results reveal that the ability of the two RNAs to be copackaged directly determines the recombination potential of two HIV-1 variants.

In the previous section we discussed that the recombination rates of gammaretroviruses appear to be lower than those of lentiviruses, although the RTs of these viruses appear to have similar abilities to perform template switching. Since the ability of the RNAs to be copackaged determines recombination potential, if gammretroviruses do not form heterozygous particles as efficiently as lentiviruses, this could explain the lower observed recombination rates. Indeed, experimental evidence suggests that RNAs derived from two gammaretroviruses may not be copackaged together as efficiently as those of two lentiviruses (HIV-1) [94]. It was found that recombination between two MLV vectors that were introduced into producer cells by cotransfection recombine more efficiently than the same two vectors introduced into cells at separate times by step-transfection [95]. As DNAs from cotransfection are more likely to be integrated close to one another than those from step-transfection, it was proposed that perhaps MLV RNA dimerizes very early during biogenesis and is more likely to pair with RNA transcribed from the same provirus, thereby promoting homodimerization and increasing the ratios of homozygous particles in the viral population. This attractive hypothesis has not been proven directly, as the proportion of the heterozygous virus population in MLV and factors that affect these proportions remain unknown.

The prerequisite for copackaging of RNA derived from two proviruses is having cells infected by more than one virus (double infection). Cell culture studies showed that double infection occurs more frequently than expected from the multiplicity of infection (moi) [96]; part of the cause for the increased double infection is that some target T cells express more receptors/coreceptors and are more susceptible to infection [97]. Double infection is more frequent via cell-mediated than the cell-free infection route (ref) as transfer of multiple viruses occurs during cell-mediated infection [96,98]. The frequency of double infected cells in HIV-1-infected patients have also been studied. In an earlier study using *in situ* hybridization, it was shown that the infected spleen cells of infected patients contain multiple copies of HIV-1 DNA [99]. A recent study used a newly developed single cell assay to study PBMCs of HIV-1 infected patients and found that most of the infected cells contained one copy of HIV-1 DNA [100]; as the infection MOI is unknown, it is unclear how frequently doubly-infected cells are expected. Thus it is difficult to compare this study with the cell culture system. However, the two studies of infected patient samples appear to point to fairly different conclusions. At this time, it is unclear whether double HIV-1 infected cells exist in spleen cells more frequently than in PBMCs or different infected individuals have different distributions of doubly-infected cells. Further studies will be needed to distinguish these possibilities.

### 3.3. Fidelity of Recombination and Its Contribution to the Overall Mutation Frequency

Retroviral homologous recombination events are generally precise because this process involves the base-pairing of the donor and acceptor template sequences as described in the dynamic copy-choice model. This is further demonstrated by the efficient reconstitution of marker genes during direct repeat deletion as discussed in the previous section. The accuracy of recombination events has also been directly examined for HIV-1. In an intersubtype HIV-1 recombination study [101], 152 recombination events were identified and their junction sequences analyzed. Although most of the recombination events were accurate (143/152), 6% of the junctions (9/152) contained errors. These erroneous events included substitutions (5/9), insertions caused by template misalignment (2/9), and deletions or substitutions likely caused by template misalignment (2/9). These 9 mutations were among the 76 mutations identified in the viral genomes in this study and occupied a significant portion of the total mutations generated. Therefore, recombination events are mostly accurate but do make occasional mistakes. Because recombination occurs very frequently during retroviral replication, these occasional mistakes can accumulate to a significant level [101].

## 4. Effects of Sequence Diversity and RNA Secondary Structures on Recombination

### 4.1. The Effects of Sequence Diversity on HIV-1 Recombination

HIV-1 has acquired extensive genetic diversity after its entry into the human population. Although variants from one subtype can dominate HIV-1 infection in certain countries, co-circulation of variants from multiple subtypes can be found in many geographic areas. Therefore, HIV-1 recombination can occur between variants with a wide range of sequence similarities. As depicted in the dynamic copy-choice model (Figure 2), stretches of homologous sequence are required between the newly-synthesized DNA and the acceptor RNA template in the annealing region for template switching to occur, and the larger the region of homology, the more likely recombination can occur (Figure 3). Using single-cycle replication assays, thereby eliminating the effects of natural selection, several studies demonstrated that increased sequence diversity produced reduced levels of recombination in different regions of the HIV-1 genome including *gag* [102], *pol* [103], and *env* [104–106]. A detailed study analyzing events in the *pol* gene in one round of replication showed that the recombination frequency between subtypes B and F (90% nucleotide sequence similarity) was ∼30% lower than that between two subtype B variants (96% nucleotide sequence similarity) [103]. Analogous conclusions were obtained in a recent similar study on *env* recombination, although the different quantification methods make a direct comparison of the results difficult [106]. Recombination in the *gag* gene in a 0.3-kb target sequence in the capsid region was also studied by detecting Gag expression using flow cytometry. Compared with recombination between variants containing identical target sequences, it was found that recombination was reduced 3-fold, and 10–13-fold when the sequence similarity was reduced to 91%, and 72–73%, respectively [102].

Recombination frequencies between HIV-1 variants from the same subtype, different subtype, and different groups have been studied using a *gfp* gene as a marker to measure recombination events. Although the target *gfp* gene sequence is identical in all of these studies, the HIV-1 genomes contain different levels of sequence similarity. Compared with the events from two variants of the same subtype, *gfp* recombination is 2-fold less in two variants from different HIV-1 subtypes containing the same DIS sequences; these variants have ∼85–90% sequence similarity [83,87]. Furthermore, the *gfp* recombination rate is drastically reduced (∼5-fold) between HIV-1 variants from different groups that contain ∼70% sequence similarity even though these variants have the same DIS sequences [102]. Therefore, sequence diversity can also indirectly reduce recombination in regions with high local homology. The exact mechanism underlying such an effect remains to be elucidated. Differences in the primary sequences might produce differences in secondary structures between distant strains that impair co-packaging or reduce RNA accessibility to RT during strand switching.

Several studies investigated the correlation between recombination hot-spots and sequence similarity in the HIV-1 genome. While one study could associate the location of hot-spots with areas of high sequence homology [107], subsequent studies indicated that although sequence diversity can produce local fluctuations in crossover frequency, it is unlikely to produce recombination hotspots [103,108–110]. The distribution of recombination breakpoints *in vivo* is more likely the result of the interplay between sequence homology, RNA structure, and RT strand switching that produce complex and different recombination patterns for each pair of HIV-1 strains.

Taken together, these results show that recombinants between highly similar strains, such as those belonging to the same subtype, are generated at the highest frequencies. More diverse viruses, such as variants belonging to different subtypes, recombine with lower frequencies. Finally, very distant HIV-1 strains, such as those classified as different groups, recombine at very low frequencies [102,103].

### 4.2. The Effects of RNA Secondary Structure on HIV-1 Recombination

RNA secondary structures have been found to be capable of producing recombination hotspots. Different groups have identified recombination hotspots associated with RNA hairpins in *gag* [111], *pol* [110] and *env* [16,112]. The mechanism regulating the effect of RNA structures on recombination is still not completely understood. The dynamic copy model predicts that stalling of the RT at the base of stem-loops, which alters the balance between DNA synthesis and RNA degradation activities of the enzyme, should increase the rate of template-switching and consequently the frequency of recombination. However, secondary structures can induce template switching events even when correlation with pause sites during reverse transcription are not observed [65]. In such instances, characterized by recombination occurring inside the stem-loop rather than at its base, a direct interaction of the hairpin structures in the donor and acceptor templates has been proposed [113]. One model proposed that the single-stranded loops of nascent DNA and acceptor RNA template could anneal and initiate the pairing of the stems, eventually producing a cross-over in the descending stem of the acceptor RNA [112,114].

Recently, the entire structure of full-length HIV-1 RNA was predicted using the selective 2′-hydroxyl acylation analyzed by primer extension (SHAPE) methodology [115]. Comparison of the RNA and genetic structure demonstrate that many RNA structures are located between domains or genes encoded in the HIV-1 genome; this observation suggests that these RNA structures may have a role in modulating the translation or folding of HIV-1 proteins. In light of the SHAPE results, it appears that most of the previously identified recombination hotspots in the *env* gene, localize to inter-domain or inter-genic junctions, in both intra- and inter-subtype recombinants [15,104,107,110–112]. Given the association between RNA stem-loops and recombination described previously, these findings raise the intriguing possibility that the identified structures have two different functions in two relevant steps of viral replication: facilitating recombination and regulating translation. A recent study showed a direct association between crossover location and local inter-domain RNA structures in the *env* gene of HIV-1 [116] when recombination was limited to one cycle of replication. Furthermore, recombination breakpoints clustered around gene junctions due to the local secondary structure even in the absence of selection. The data obtained for the *env* gene strongly supports a role for RNA structures in recombination. Although the effect of the RNA structures on the recombination in the *env* gene is convincing, whether it can be applied to the entire HIV-1 genome is yet to be determined. For example, using a single cycle assay, the junctions of intra- and intersubtype recombination in the *pol* gene have been studied; when compared with the predicted structures from the SHAPE study, there is no association between RNA structures and breakpoints distribution [103]. However, stem-loops predicted in *env* are generally more prominent than those in *pol* [115]. It is possible that the different results obtained in the two regions in the HIV-1 genome are simply caused by the extent of the secondary structures, and only RNA structures with sufficient size or stability can affect recombination, whereas minor structures have no effect or only subtle effects that were not detected in these studies.

## 5. Fitness of the Progeny Recombinant

Recombination assorts polymorphisms in retroviral genomes. One of the consequences of this process is that the linkage of the co-evolved sequences may be disrupted in many recombinants. Therefore, although recombination increases diversity of the viral population, many of the newly generated recombinants may have altered or reduced replication fitness than their parents, which may predict that they will have a different probability to survive. Using different experimental approaches, two recent papers addressed the fitness of the newly generated recombinants. In one study, the recombination junctions in the *env* gene were determined and the functions of a set of recombinant Envs were tested using a single cycle assay [106]. It was shown that viruses with these recombinant Env proteins had varied abilities to replicate; although some recombinant Env proteins could generate viral titers similar to those of the parents, some recombinant Env proteins clearly generated far lower titers than those of the parental Env proteins. Comparing the functions of these Env and recombination junctions of the circulating strains suggests that the function of the Env proteins is an important factor for the survival of the recombinants [106].

In another study, the patterns of the observed recombinants in the *pol* gene were compared with or without the selection pressure on the Pol proteins [103] by analyzing recombinants generated in a single round assay and in a multiple round assay. Regardless of the requirement for Pol function, the recombination patterns between two subtype B variants were very similar; recombination junctions were observed throughout the *pol* gene. These results indicate that recombination between strains with high sequence similarity are likely to result in genomes that are functional; therefore, the distribution of crossovers is unaffected in the recombinant population regardless whether Pol function is selected. However, a very different picture emerged in the studies of recombination between subtypes B and F. When the Pol function was not required, recombination junctions were observed throughout the *pol* gene; in contrast, when the Pol function was selected, recombination junctions were observed in only a few clustered regions in the *pol* gene. These results revealed that although recombination between divergent strains such as subtypes B and F can occur throughout the *pol* gene, most of the recombinants did not survive multiple rounds of replication in which the Pol function was required.

Taken together, both of these studies revealed that viral replication fitness is an important factor for the survival of the recombinants; many newly generated recombinants, especially those from divergent variants, have altered replication fitness and face a strong purifying selection. These studies also suggest that despite the frequent observation of the circulating recombinant strains (see the following section for detail), there are many more recombinants that are generated but are lost as a result of the purifying selection.

## 6. Recombination in the HIV-1 Epidemics

### 6.1. Intersubtype HIV-1 Recombinants: Evidence of Recombination-Generated Variation in Current HIV- Isolates

One of the best examples of how recombination can generate diversity in a virus population is demonstrated by the observation of HIV-1 intersubtype recombinants. Although recombination occurs frequently with variants in the same subtype, these recombination events are often difficult to identify unless the parental viral sequences are known. In contrast, the genetic distance between different group M subtypes makes it easier to identify intersubtype recombinants. The increased co-circulation of different subtypes of HIV-1 in many areas of the world has fostered the generation of a high number of recombinant forms [3,117–119]. Several such circulating recombinant forms (CRFs) have become important in the global epidemic [2,120]; to date, 49 CRFs have been classified [121] in the HIV-1 pandemic. In addition, a large number of unique recombinant forms (URFs) have been detected in the infected population. Some of the early identified CRFs have become predominant in their area of origin, probably due to either their early introduction or to fitness advantage [119]. For example, CRF01_AE, a recombinant between subtype A1 and the putative subtype E, is the predominant strain in South-East Asia [122]; CRF01_AG, a variant which has been recently proposed to be in fact the parental subtype G rather than a recombinant [123], is the dominant form in West and Central Africa [120]; and CRFs 07 and 08, which are B/C recombinants that originally appeared among drug-users in China, have now spread to a vast portion of the infected Chinese population [124].

The genetic composition of recombinants usually reflects the level of co-circulation of different strains in a given geographical area. B/F recombinants such as CRFs 12, 17, 28, 29, 38, 39, 40, 44, and 46, are widely spread in South America, where they represent a high percentage of infections. B/G recombinants such as CRFs 14, 20, 23, and 24, have been identified mostly in Cuba, Spain and Portugal [125–127], while recombinants involving the CRF01 (CRFs 15, 33, 34, and 48) are mostly common in South-East Asia. On the other hand, most of the CRFs classified so far have been isolated from Central-African patients, due to the extensive co-circulation of different genetic forms in their area of origin [119]. Overall, the most recent estimates place the prevalence of recombinant forms at about 20% of infections worldwide; these surveys also indicate that non-B subtypes and recombinants have been stably introduced into areas previously restricted to infection by the subtype B variants such as North America and Western Europe [3,128]. The possible impact of recombination on the evolution of escape mutants and drug resistant strains has raised concern in the medical community about long term outcome of antiviral treatment and the effects on patient management.

### 6.2. Detection, Replicative Fitness, and Drug-Resistance of the Intersubtype Recombinants

Most antivirals and detection assays commercially available for HIV-1 have been developed and tested using subtype B strains. The performance of most HIV-1 RNA quantification assays in identifying subtypes different from B are variable between different assays and usually lower compared to subtype B detection [129,130]. As a consequence, depending on the subtypes involved in each recombinant and the patterns of recombination, the levels of circulating virus could be undetectable or grossly underestimated by the currently available kits [131]. Plasma level of viral RNA is an important parameter for determining drug treatment and assessing the efficacy of therapy [132,133]; unreliable RNA determination could have detrimental effects on patient prognosis and treatment outcome.

Although data are available for a limited number of subtypes, HIV-1 variants demonstrate a range of different replicative fitnesses [134]. Thus, it is possible that intersubtype recombinants may acquire a higher fitness than a parental strain; indeed, CRF02_AG was hypothesized to have had such a gain of fitness, thereby allowing this recombinant form to be a predominant variant in West and Central Africa [135,136]. Similarly, subtype B variants were introduced to South-East Asia before CRF01_AE entered this region through the intravenous drug user population [137], but this recombinant quickly became the predominant form in all risk categories [122] possibly due to higher infectivity of the recombinant form [138]. Despite the differences in replicative fitness, direct analyses of diverse HIV-1 strains only detected significant differences in the viral pathogenicity of subtype D infection [139–141], indicating that further studies are needed to clarify the link between replicative fitness and pathogenesis in intersubtype recombinants.

Most of our current understanding of mutations associated with drug resistance and their mechanisms are from studies based on subtype B variants. However, it is known that several non-B subtype variants carry polymorphisms associated with low level of drug resistance in subtype B variants [142,143]. Such sequence polymorphisms can produce different responses to specific drugs or classes of drugs in some HIV-1 variants and are associated with various drug-resistance evolutionary pathways [144]. Furthermore, recent studies showed that although most HIV-1 strains remain susceptible to antiretroviral drugs [145–147], variants from different HIV-1 subtypes can have intrinsic differences in the level of resistance to antiretrovirals [148]. Recombination offers the potential to bring together existing resistance mutations, or beneficial molecular features of intrinsically-resistant enzymes [149], thereby generating progenies with more selective advantages than their parents. Indeed, mathematical modeling studies demonstrated that by reducing the impact of linkage disequilibrium, recombination allows a faster reassortment of mutations and a higher adaptation rate [150].

### 6.3. Recombinants and Variation Create Challenges to HIV-1 Vaccine Development

Vaccination is the most effective way of preventing viral diseases. However, the extensive genetic variability and the diverse assortment of antigen epitopes associated with HIV-1 strains create a major challenge for the development of a successful AIDS vaccine. During early infection, HIV-1 evolution is driven by selection and fixation of mutations conferring escape to cytotoxic T-cell responses. HIV-1 variants carrying escape mutations can be selected for in the infected individual, or can be acquired directly through the chain of transmission [151]. Since the initial transmission in an individual most often involves between 1–3 infection events [152], recombination between founder viruses can facilitate escape from cytotoxic T-cell responses by allowing re-assortment of mutations escaping immune response during early HIV-1 infection. The effectiveness of recombination in epitope shuffling has been examined in recent studies of patients infected with more than one strain of HIV-1. It was shown that by the time the first anti-HIV antibody was detected, most of the circulating viruses were already recombinants [153]. Additionally, a recent case study showed that rapid recombination occurred after superinfection between the existing virus and the newly transmitted virus to generate recombinants containing different epitopes in Gag and Env to avoid the pre-existing cytotoxic T cell response. The generation of escape variants caused the failure of the immune response to control HIV-1 replication and viral loads escalated in the patient [154]. Furthermore, in non-human primate models, recombination between the live attenuated SIV vaccine and pathogenic strains of SIV has resulted in breakthrough viremia and the generation of more virulent strains of SIV [155–158]. These studies highlight the complications that can be caused by recombination events in the development of a successful HIV-1 vaccine.

For a vaccine to be effective on a population level it must elicit sterilizing immune responses that recognize a majority of the circulating strains of HIV-1. Efforts to overcome the problem of HIV-1 diversity include strategies designed to achieve high epitope coverage. One approach uses polyvalent mosaic immunogens derived by *in silico* recombination of natural strains of HIV. These so-called mosaic vaccines are intended to induce cellular immune responses that recognize genetically diverse virus isolates [159,160]. Another strategy is to design epitopes using the center of the tree (COT) approach [159,160]. Briefly, a COT sequence is calculated from a phylogenetic tree of different HIV-1 isolates, by re-rooting the tree at the point that describes the least-squares distance to all the tips on the tree. In this way a majority of the variety of the sequences in the tree can be included in a single epitope [159,160]. Two recent studies have demonstrated that such mosaic HIV-1 vaccines appear to increase the breadth and depth of cellular immune responses in Rhesus monkeys [161,162]. Whether these strategies will also be effective in preventing HIV-1 infection in humans remains to be demonstrated.

The impact of recombination on HIV-1 detection, pathogenicity, treatment, and vaccine design can have important consequences on patient management and therapy outcome. Most studies investigating these issues are focused on “pure” non-B subtypes, underlining the need for data regarding recombinant forms. Given the increasing global prevalence of recombinants, a better understanding of their biology *in vivo* would help in devising optimal treatment strategies and development of a vaccine.

## 7. Conclusions

Retroviruses have one of the highest recombination rates among all viruses. Frequent recombination reassorts viral sequences to generate variants containing different combinations of polymorphic sequences, thereby generating high diversity in the viral population, which improve the odds that some variants in the population can survive the ever changing selection pressure in the environment. There are clear examples of such advantages in the replication of HIV-1. For example, recombination can generate a variant to escape the host cell immune response; similarly, recombination can also assort existing drug-resistance mutations to generate a more resistant virus or a variant that is resistant to more than one drug. Additionally, by assorting sequences, recombination can also preserve diversity outside the selected region. Recombination is a major driving force for viral diversity; understanding the mechanisms and the factors that affect recombination can ultimately help us to develop better treatment and a preventative vaccine to control the AIDS epidemic.
